# Review on Bioenergy Storage Systems for Preserving and Improving Feedstock Value

**DOI:** 10.3389/fbioe.2020.00370

**Published:** 2020-04-28

**Authors:** Lynn M. Wendt, Haiyan Zhao

**Affiliations:** ^1^Environmental Science Program, University of Idaho, Idaho Falls, ID, United States; ^2^Department of Biological and Chemical Science and Engineering, Idaho National Laboratory, Idaho Falls, ID, United States; ^3^Department of Chemical and Materials Engineering, University of Idaho, Idaho Falls, ID, United States

**Keywords:** biomass, biofuels, feedstock logistics, long-term storage, recalcitrance

## Abstract

Long-term storage is a necessary unit operation in the biomass feedstock logistics supply chain, enabling biorefineries to run year-round despite daily, monthly, and seasonal variations in feedstock availability. At a minimum, effective storage approaches must preserve biomass. Uncontrolled loss of biomass due to microbial degradation is common when storage conditions are not optimized. This can lead to physical and mechanical challenges with biomass handling, size reduction, preprocessing, and ultimately conversion. This review summarizes the unit operations of dry and wet storage and how they may contribute to preserving or even improving feedstock value for biorefineries.

## Introduction

The utilization of renewable biomass feedstocks for fuel and energy production offers the potential to displace a significant portion of petroleum-based transportation fuels and related greenhouse gas emissions. The transportation sector utilizes one third of all energy and 70% of all petroleum consumed in the United States (Davis and Boundy, 2019). Electrification of the grid with renewable energy sources, such as wind and solar power, will contribute to reducing carbon-based fuels in the light-duty vehicle fleet. However, the need for sustainably-produced, liquid transportation fuels will remain since aviation fuel use is projected to double in the next 20 years (International Air Transport Association, 2018) and heavy-duty vehicles and marine vessels will likely require carbon-based fuels (U.S. Department of Energy and Bioenergy Technologies Office, 2016). Furthermore, bio-derived fuel and chemical production can result in the carbon negative technologies that are necessary to counteract the global warming of 1.5°C above pre-industrial levels (First, 2019).

Renewable biomass feedstocks include non-food material such as corn stover, herbaceous and woody energy crops, forest product residues, algae, and municipal solid waste. Estimates suggest that over 1 billion tons of these feedstocks are available annually for sustainable utilization in bioenergy production systems (Langholtz et al., 2016). This bioeconomy has the potential to create over 1 million jobs and $260 billion in U.S. revenue, displace 30% of liquid transportation fuels, and reduce 50% of greenhouse gases compared to petroleum (U.S. Department of Energy and Bioenergy Technologies Office, 2016).

**FIGURE 1:**
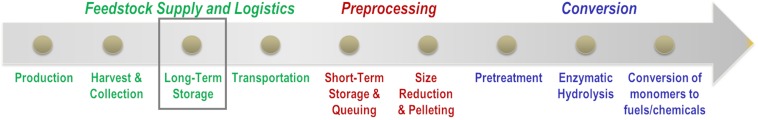
Unit operations in the conversion of lignocellulosic biomass to fuels and chemicals through a biochemical conversion approach. This review will describe the impact of long-term storage (gray box) on conversion operations.

Major unit operations in the conversion of biomass feedstocks to fuels include supply and logistics operations including harvest, collection, transport, storage, and formatting followed by biochemical conversion of carbohydrates to fuels and chemicals (Figure 1). Feedstock supply and logistics unit operations generally begin with the harvest of a crop or a portion of the crop that is cultivated either on an annual basis (e.g., corn, wheat, sorghum, etc.), on a perennial basis (e.g., switchgrass, miscanthus, etc.), or a multi-year basis (e.g., willow, pine, etc.). In the case of agricultural residues including corn stover, commonly accepted practices are based on dry, baled logistics systems. Harvesting of the grain fraction of the plant is performed simultaneously or just preceding harvesting of the biomass residue (Birrell et al., 2014). Formation of windrows occurs either during harvest or by a windrower followed by drying in-field to facilitate stable storage conditions and collection of the biomass from windrows into bales (Hess et al., 2007; Shah and Darr, 2016). Bales are stored either field-side or at a centralized location until further use (Darr and Shah, 2012). Size reduction to meet biorefinery particle size specifications is performed either at the biorefinery gate (Hess et al., 2009a) or at a biomass feedstock depot (Hess et al., 2009b). Depot concepts have been proposed to facilitate densification of biomass into low-moisture pellets for stable storage and low transportation costs. The cost and performance of these logistics systems and associated unit operations have been well-documented (Hess et al., 2007, 2009a,b), and estimates in 2018 suggest that delivered cost of corn stover to a refinery is estimated at $84/US ton depending on the harvest method and the draw ratio of the biorefinery (Roni et al., 2018). These costs are low compared to the forage industry but are necessary to be competitive with fossil-based fuels of approximately $3/gallon.

Multiple approaches to convert biomass resources to energy sources exist and are generally characterized as either biochemical or thermochemical. Each conversion technology has advantages and disadvantages in terms of their flexibility to feedstock source and related chemical composition as well as regarding the product generated from that feedstock. These diverse conversion approaches facilitate utilization of geographically localized biomass feedstocks. For example, agricultural residues are concentrated in the middle and eastern portion of the U.S., while woody biomass and forest thinnings are concentrated in the southeast and western portions of the U.S. (Langholtz et al., 2016). All these conversion approaches have a role in the formation of a stable bioeconomy and reducing the dependence on fossil-fuel based resources (U.S. Department of Energy and Bioenergy Technologies Office, 2016).

Biochemical conversion of lignocellulosic biomass including corn stover has been facing technical challenges during scale up despite significant investment by three commercial-scale integrated cellulosic-based biorefineries in the U.S. last decade. All these biorefineries have struggled to make biofuels a reality. Dale summarized two primary challenges that were faced including the lack of understanding of how to stably store biomass for long durations and the difficulty to chemical deconstruction in biomass during pretreatment operations (Dale, 2017). The first challenge is a result of the susceptibility of biomass to microbial or physical loss when not stored in a stable manner, and the later issue stems from the variations and complexities in corn stover and associated challenges of converting this feedstock into fuels (Richard, 2010; Dale, 2017). Understanding lignocellulosic biomass and overcoming the associated recalcitrance is key to addressing the challenges for biochemical conversion. Therefore, the focus of this review article is aligned closely with biochemical conversion approaches for corn stover but may have applicability toward thermochemical conversion and other lignocellulosic biomass as well. This review will highlight the impact of long-term storage on conversion operations with the focus of how storage systems may be used to overcome both the challenge of stable storage for bioenergy systems and be complementary to pretreatment systems.

## Lignocellulosic Biomass Structure and Associated Recalcitrance

**FIGURE 2:**
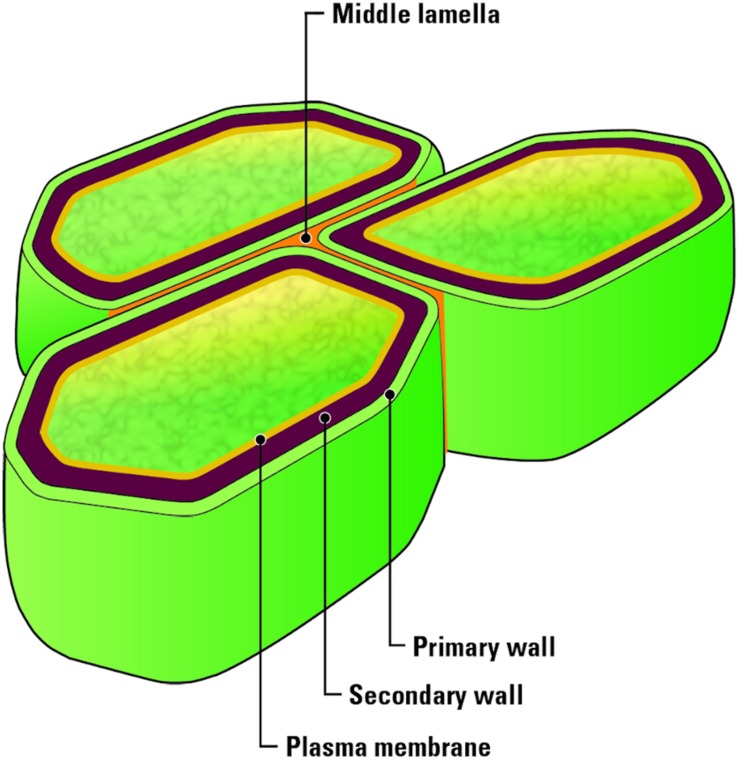
Biomass plant cell wall structure. Photo credit: U.S. Department of Energy Genomic Science Program. https://genomicscience.energy.gov.

A fundamental understanding of the structure of lignocellulosic biomass is necessary for the prediction of how biomass may be affected during each unit operation between harvest and conversion. Lignocellulosic biomass, such as corn stover, consists of an intricate combination of cellulose, hemicellulose, and lignin, that provide strength to the plant cell walls (Cosgrove, 2005; Cosgrove and Jarvis, 2012). Plant walls (Figure 2) consist of a primary wall, which is composed of cellulose, xyloglucans, and pectin as well as 10–20% protein (Himmel et al., 2007). Secondary walls contain cellulose, xylans, glucomannans and lignin and are separated into S1, S2, and S3 layers (Mellerowicz and Sundberg, 2008). A thin layer, termed the middle lamella, connects plant cells to each other and is rich is pectin (Iwai et al., 2002). These cell wall components are multi-functional, supporting nutrient transport during growth while providing strength to the plant such that it can withstand environmental factors including wind, moisture, and physical impact. However, the complex nature of biomass tissues and their chemical makeup presents a challenge for a biorefinery. The term recalcitrance describes the resistance of lignocellulosic biomass to biological, chemical, and thermal methods of deconstruction. Each plant tissue and cell wall layer are built of unique chemical signatures increasing this recalcitrance to deconstruction, and an understanding of the chemical makeup and bonds holding them together is essential in order to effectively deconstruct and depolymerize lignocellulosic biomass.

**FIGURE 3:**
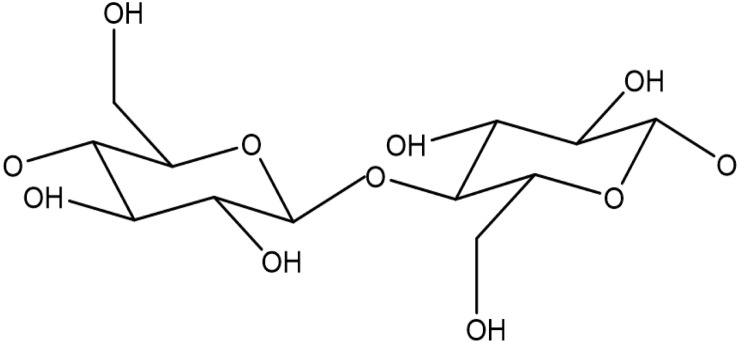
Cellulose backbone consisting of D-glucose molecules linked in the β-1,4 position and rotated 180 degrees from each other.

Cellulose microfibrils are the main component of the primary and secondary cell wall in plants. Microfibrils are composed of multiple glucose chains arranged in parallel in a crystalline fashion, with individual glucose chains linked internally and to each other through hydrogen bonds (Himmel et al., 2007). Individual glucan chains and are comprised of 500–14,000 repeating D-glucose units; two D-glucose molecules are linked in the β-1,4 position and rotated 180 degrees from each other, forming a cellobiose unit as shown in Figure 3 (Mohnen et al., 2009). Himmel has proposed that cellulose microfibrils are arranged into 36 glucan chains arranged in a radial fashion (Himmel et al., 2007), whereas Fernandes has proposed 18–24 glucan chains in sheets are present in each microfibril (Fernandes et al., 2011). Primary cell walls contain only three to four layers of the microfibrils, while the secondary cell walls are thought to contain hundreds of microfibrils (McCann et al., 1990). One distinct attribute of secondary cells walls is the varied orientation of cellulose microfibrils in the S1, S2, and S3 layers, which contributes to the strength of the plant tissues (Zhong and Ye, 2014).

Hemicellulose is comprised of a complex matrix of polysaccharides generally consisting of long chains with a β-1,4-backbone and multiple side chains. Hemicellulose surrounds cellulose microfibrils and associates with them through hydrogen bonds (Busse-Wicher et al., 2014), helping to strengthen the plant’s primary and secondary cell walls (Marriott et al., 2016). The composition and complexity of hemicellulose has been extensively reviewed (Mohnen et al., 2009; Marriott et al., 2016). Xyloglucan has a -1,4-glucan backbone with xylose side chains. Xylans have a -1,4-xylose backbone and can contain other polysaccharide side chains including arabinan and glucuronic acid. Mixed-linkage glucans are linked at both -1,3 and -1,4 positions. Gluco- and galactomannans consist of a -1,4 mannan backbone that can be substituted with glucan and galactan, respectfully. Acetyls and phenolic acids, such as ferulic acid, are common side chains linked to the hemicellulose (Harris and Picataggio, 2008), and these have been shown to reduce the accessibility of cellulose to enzymatic attack (Selig et al., 2015). Therefore, the association of hemicellulose and cellulose is a key factor in reducing biomass recalcitrance.

**FIGURE 4:**
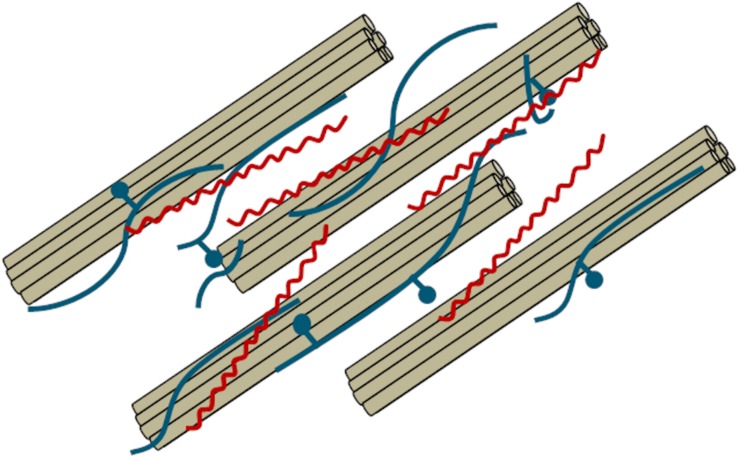
Graphical description of cellulose microbifibrils (brown) surrounded by hemicellulose (blue) and pectin (red). Adapted from Cosgrove, 2005.

Pectin is a 1,4-linked galacturonic acid-based polysaccharide that is principally located in the middle lamella and primary cell wall of lignocellulosic biomass (O’Neill et al., 1990; Mohnen et al., 2009). Pectin is generally not located in the secondary cell wall but can be present in the outer secondary cell wall layers. Pectin is proposed to form covalent bonds with hemicellulose and increases the strength of the cell wall (Popper and Fry, 2008). A graphical depiction of the interactions between pectin (red) with hemicellulose (blue) and cellulose (brown) is shown in Figure 4. Pectin content is generally highest in dicots but is also present in monocots (Jarvis et al., 1988). Pectin can act as a barrier against enzymatic attack and therefore is an important component when considering the conversion of lignocellulosic biomass to biofuels.

**FIGURE 5:**
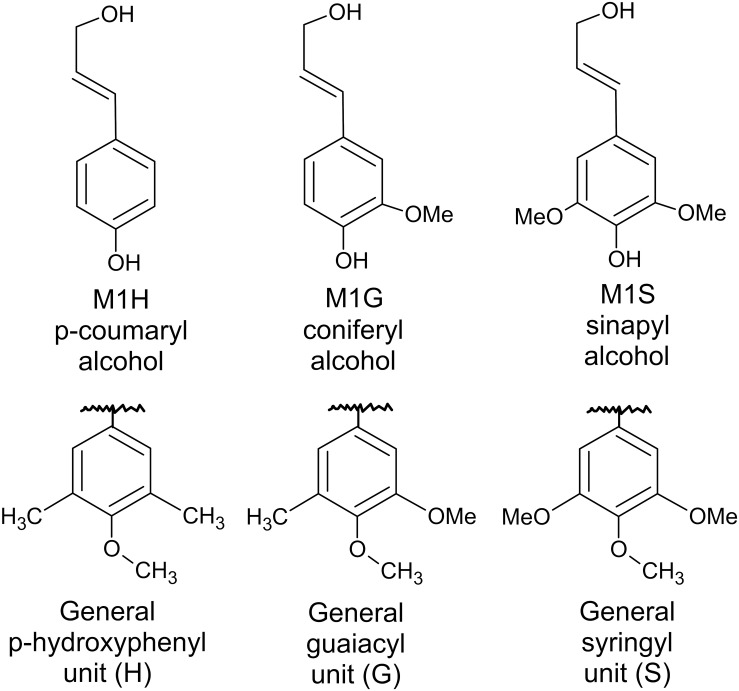
Lignin alcohol precursors and resulting monolignins.

Lignin is a complex molecule that is made up of hundreds of monomers. Lignin concentrations are highest in middle lamella and primary cell walls (Donaldson et al., 2001), yet these components are of low concentration in the cell wall compared to secondary cell walls. Lignin is also present in the cellulose microfibril-rich secondary cells walls (Freudenberg and Neish, 1968) of which the S2 layer is the largest fraction (Mellerowicz and Sundberg, 2008). Lignin fills the space between cellulose, hemicellulose, and pectin and thus serves to strengthen the cell wall. Lignin is hydrophobic and can protect the cells from enzymatic attack and resulting degradation. Monolignins are the building blocks of lignin; they are synthesized from phenylalanine in the cytosol through a complex set of enzymatic reactions and are characterized by their number of methoxy side chains (Boerjan et al., 2003). *p*-coumaryl, coniferyl, and sinapyl alcohols have zero, one, and two methoxy side chains, respectfully (Freudenberg and Neish, 1968). These monolignins are transported into the cell wall, where they are then polymerized oxidatively to another monolignin or a growing lignin chain, likely as a result of a peroxidase or laccase that results in the formation of a free radical (Boerjan et al., 2003; Ralph et al., 2004). Therefore, *p*-coumaryl, coniferyl, and sinapyl alcohols result in the formation of p-hydroxyphenyl (H), guaiacyl (G), and syringyl (S) units within a lignin molecule (Figure 5). Linkages between cellulose, hemicellulose, pectin, and lignin can be ester or ether and can either directly link these molecules or use acid bridges such as ferulic acid or hydroxycinnamin acid (Harris, 2009; Marriott et al., 2016).

The complex nature of the composition and associated bonds between cellulose microfibrils, hemicellulose, pectin, and lignin and resulting heterogeneity of plant tissues present a challenge for conversion of their respective monomers to fuels and chemicals (Himmel et al., 2007; Marriott et al., 2016). Additionally, factors such as the presence of waxes, the abundance of sclerenchyma and associated tissue strength, and inhibitors to fermentation (i.e., acetic acid, ferulic acid) contribute to biomass recalcitrance (Himmel et al., 2007).

## Lignocellulosic Biomass Conversion Approaches

Effective biochemical-based strategies for converting the biomass into fuels and chemicals generally involve the utilization of chemicals, heat, and enzymes to break down the lignocellulosic biomass into monomers followed by conversion to fuels through approaches including fermentation (Foust et al., 2009). Recalcitrance is a significant challenge for biochemical-based conversion approaches as the cellulose microfibril is not accessible to enzymatic attack until hemicellulose and lignin have been decoupled from the matrix (Himmel and Picataggio, 2009). Enzymatic action on cellulose microfibrils is further complicated by the strong hydrogen bonding within cellulose sheets in the microfibrils (Nishiyama et al., 2002) as well as the hydrophobic layer on the outside of the sheets that reduce the effectiveness of acid attack (Matthews et al., 2006).

Biochemical approaches to the conversion of lignocellulose begin to overcome this recalcitrance in a pretreatment step that utilizes the combination of temperature, caustic, and time to increase the digestibility of lignocellulosic biomass. The particle size necessary for biochemical conversion depends on pretreatment chemistry (Vidal et al., 2011), but a nominal 6 mm size is often recommended to minimize the cost of size reduction while increasing the surface area for pretreatment (Foust et al., 2009; Humbird et al., 2011). Dilute acid pretreatment generally occurs between temperatures of 140 and 200°C, and hemicellulose hydrolysis is the primary mode in which this pretreatment chemistry makes cellulose more accessible to enzymatic attack (Torget et al., 1991). Alkali treatments include applying sodium hydroxide (Grohmann et al., 1989) as well as lime (Kim and Holtzapple, 2005) to remove acetyl groups from xylan and remove lignin through oxidation (Katahira et al., 2016). Steam explosion can be used to increase the surface area though defibrillation and is catalyzed by the removal of acetyl groups from hemicellulose (Saddler et al., 1993). Ammonia-based pretreatment such as ammonia fiber explosion (AFEX) impregnates plant cells during a pressure change, which results in both deacetylation as well as reduced crystallinity of cellulose (Gollapalli et al., 2002). Ionic liquids solubilize cellulose, hemicellulose, and lignin, which are then selectively precipitated to isolate these components (Heinze et al., 2005; Shill et al., 2011). The commonality of these pretreatment methods is that they target specific biomass components with the goal to make others more accessible to subsequent enzymatic attack.

Enzymatic hydrolysis succeeding pretreatment is performed using glycosidases including cellulases or mixtures of enzymes that attack components in hemicellulose (e.g., xylanases, mannanases, arabionfuranosidases, and pectin lyases) (Bayer et al., 2004). Upon release of carbohydrate monomers, fermentation can proceed by yeast or bacteria. Ethanol fermentation was one of the first commercialized approaches for fuel generation from lignocellulosic biomass (Humbird et al., 2011) and is based on the technology of the grain ethanol industry. Additional fermentation approaches that have gained recent attention include production of carboxylic acids including butyric acid (Nelson et al., 2017; Saboe et al., 2018) or propionic acid (Wang et al., 2017) that can be upgraded catalytically to hydrocarbon fuels (Cortright et al., 2002). Succinic acid is also a produced through fermentation (Song and Lee, 2006; Salvachúa et al., 2016) and is a valuable chemical building block (Song and Lee, 2006; Nikolau et al., 2008). The commonality between all these approaches is the production and subsequent utilization of carbohydrate monomers to higher-value fuels and chemicals.

Recent attention has also been focused on lignin utilization to increase the economics of biorefineries. Combustion for process heat was the original use of lignin in cellulosic biorefinery models (Humbird et al., 2011). However, the pressure for lignocellulose-derived fuels to be cost competitive with fossil-based transportation fuels require either lower conversion costs or higher value end uses of the conversion products. Lignin can be depolymerized by chemicals and enzymes and utilized for high value products (Ragauskas et al., 2014). Multiple fermentation pathways exist for lignin monomers including adipic acid (Vardon et al., 2015) and muconic acid (Salvachúa et al., 2018). Improvements in biomass recalcitrance reduction are also necessary to further advance this field given the complexity of lignin molecules.

Thermochemical approaches for biomass conversion utilize heat and/or catalysts to create either heat through combustion, into liquids such as bio-oils through pyrolysis of liquefaction, or into combustible gases through gasification (McKendry, 2002). Thermochemical conversion approaches have been extensively reviewed elsewhere. Thermochemical approaches require biomass to be at a small particle size to increase surface area, typically less than 2 mm. Thermochemical conversion is often favorable for soft and hardwood biomass feedstock due to their elevated lignin level compared to herbaceous biomass feedstocks since lignin has a higher calorific value compared to carbohydrates. Thermochemical conversion approaches also can be used to generate combustible gases from low value feedstocks such as municipal solid wastes. Biomass recalcitrance in relation to thermochemical conversion but is gaining attention in order to understand mechanisms that improve fuel yield (McCann and Carpita, 2015). For example, Kim et al. reported on the application of partial-oxidative pyrolysis to depolymerize lignin and thus allow for increased conversion of cellulose to levoglucosan in bio-oil (Kim et al., 2014). Similarly, a low temperature pyrolysis method combined with two-dimensional gas chromatography coupled with mass spectrometry has been shown to identify storage related changes in cellulose, hemicellulose, and lignin-based pyrolysis products (Groenewold et al., 2019). Advancements in the understanding of biomass recalcitrance and related yield in thermochemical conversion systems is necessary to further predict approaches to increase fuel yield.

## Biomass Storage Systems

Seasonal variation is a challenge for most agricultural products, necessitating storage in order to provide a biorefinery with year-round access to the product. Agricultural residues, such as corn stover, are typically available during a 1–2-months window and are dependent on the harvest of the primary product. Energy crops are also harvested seasonally but have a more flexible harvest window since it is the primary product as opposed to residues that are reliant on a commodity crop. Engineered storage systems offer the opportunity to minimize the seasonal variation of biomass availability and allow a biorefinery to operate year-round with a consistent feedstock supply. Long term storage also allows for a biorefinery to be sized at the appropriate scale such that down-time is minimized, and this reduces costly capital expenditures.

### Dry Storage Systems

**FIGURE 6:**
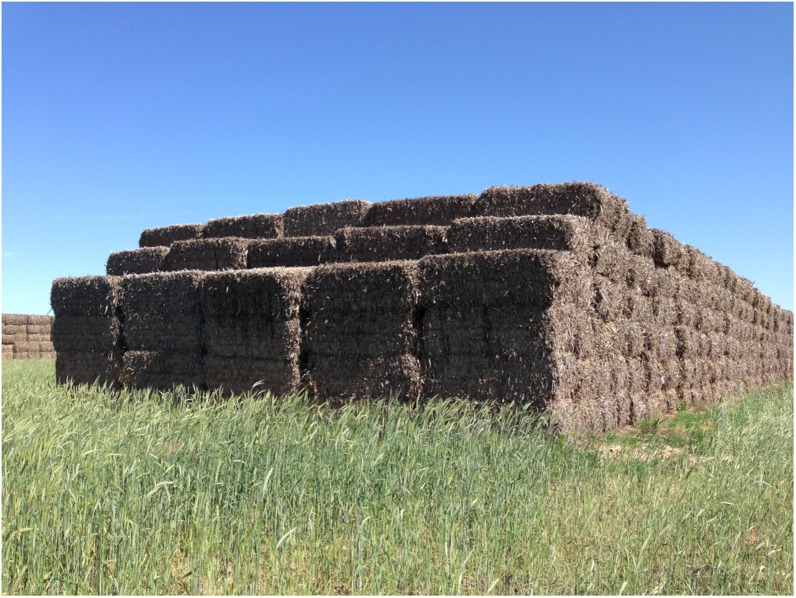
Corn stover bale stack at a satellite storage location.

The primary goal in storage is to preserve the reducing equivalents in biomass, and dry storage systems are one solution for stably storing biomass over long periods of time. Bale stacks are the state of technology for field-side storage of agricultural residues (Shah et al., 2011), and a corn stover bale stack is shown in Figure 6. Bales are generally covered with tarps to reduce moisture accumulation from precipitation, while improved surfaces are recommended to prevent wicking of soil moisture by the bottom bales. Smith et al. described the moisture distribution of tarped and untarped corn stover bales entering storage at the same moisture content (22% wet basis); after 5 and 9 months moisture had redistributed to levels up to 65% just under the surface of the tarp as well as in the bottom bales where moisture adequate drainage was not present (Smith et al., 2013). Overall, bale-based storage can effectively preserve biomass when under ideal conditions but must be managed carefully to maintain stable conditions.

Biomass stored in dry systems is particularly susceptible to microbial degradation if conditions conducive to enzymatic activity or microorganism growth are present. Water activity (a_w_), which describes the ability of water to react chemically and biologically, drives the storage stability of a range of industrially-relevant nutritional products for human and livestock consumption (Beuchat, 1981). Water activity ranges between 0 and 1 and corresponds with no water being available for utilization and all water being available, respectfully. Water activity can be calculated by determining the relative humidity of air in a sample in equilibrium, and moisture sorption isotherms are used to determine the relationship of water activity and moisture content for a given material (Beuchat, 1981). Water activity is also impacted by temperature, which is one reason refrigeration is an effective preservation method. The relationship between water activity and microbial stability is well-documented, with bacteria growth prevalent when a_w_ > 0.85, yeasts prevalent between a_w_ values of 0.80–0.90, and mold growth dominant when a_w_ value is 0.85–0.60. Only enzymes are considered active at a_w_ < 0.60. Athmanathan et al. (2015) related water activity to dry matter loss in switchgrass and demonstrated no appreciable loss at a_w_ > 0.85, which corresponded to a moisture content of approximately 16% (wet basis). The relationship between biomass source, chemical composition, free versus bound water, and environmental conditions such as temperature can be used relate moisture content and water activity, and an enhanced understanding of these parameters can be used to positively impact biomass storage stability.

A recent study suggests that an average of only 36% of corn stover harvested in the U.S. is capable of entering long-term bale storage at moisture levels that result in stable storage (Oyedeji et al., 2017), which makes corn stover a particularly challenging feedstock to store using dry approaches. Similarly, a moisture content of 20% or less has been recommended for stable corn stover in baled storage (Darr and Shah, 2012). Significant losses of dry matter have been reported in field-side storage of corn stover that exceeds this moisture threshold due to microbial degradation (Shinners et al., 2007; Smith et al., 2013). Microbial degradation of aerobically stored biomass materials can be characterized in terms of CO_2_ production, microbial heat generation and resulting temperature increase, and dry matter loss (McGechan, 1990; Wendt et al., 2014). Aerobic microbial degradation by bacteria, yeast, and fungi consumes valuable carbohydrates and produces CO_2_ as a byproduct, leaving behind material enriched in non-fermentable biomass components such as ash. This degradation has been documented to begin with hydrolysis of acetyl groups and reduction in hemicellulose, which has been measured by wet chemical analysis, such that the microorganisms can access cellulose (Wendt et al., 2014, 2018a). Hemicellulose modification has also been documented in corn stover that suffered severe aerobic degradation during storage using a pyrolysis/two-dimensional gas chromatography/mass spectrometry (Py-GCxGC-MS) approach (Groenewold et al., 2019). In this study, formation of acetic acid and furfural, which correlate to acetyl and C5 sugar degradation, was increased in corn stover samples that suffered severe degradation compared to samples that suffered only mild degradation. Understanding how microbial degradation might be used as a partial pretreatment is a topic that has not been widely reported, and this moisture management approach may have applicability in bioenergy systems that rely on dry storage approaches.

Bale storage systems can be cost prohibitive in many industrial settings because the shear amount of combustible material present must be managed safely. Corn stover bales are at risk of loss due to fires (Webb et al., 2018), necessitating significant land use to create a physical barrier to protect a burning stack from igniting other stacks. Additional insight into how dry storage systems can be managed and/or configured to reduce this risk in a cost-effective manner will support bioconversion designs by protecting the valuable asset of biomass in the logistics supply chain.

### Wet Storage Systems

**FIGURE 7:**
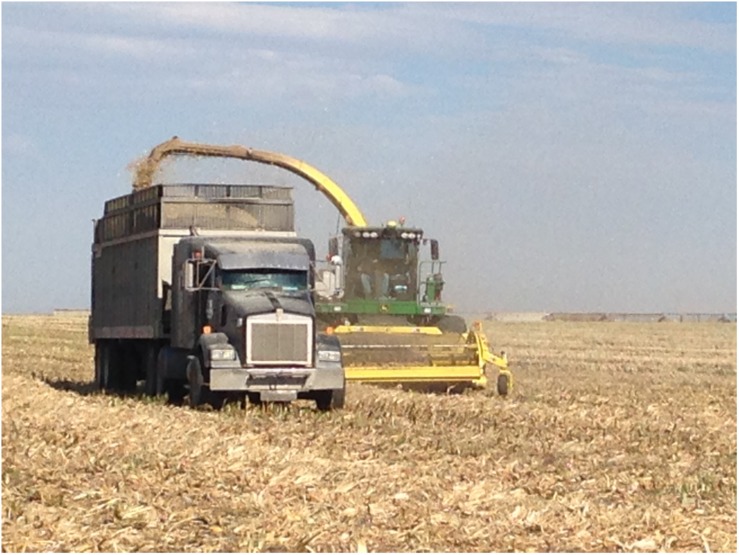
Collection of corn stover with forage chopper into a walking floor trailer.

**FIGURE 8:**
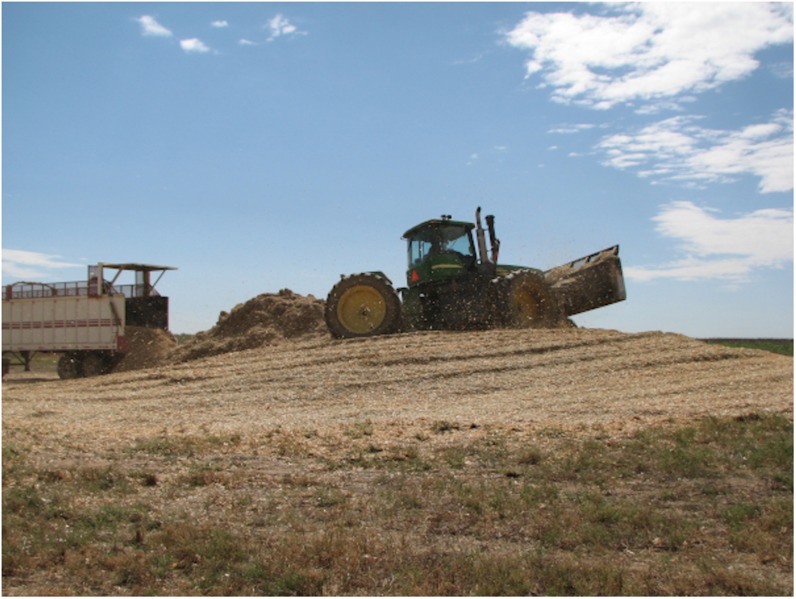
Simultaneous formation and compaction of a drive over storage pile with corn stover unloaded from walking floor trailers.

**FIGURE 9:**
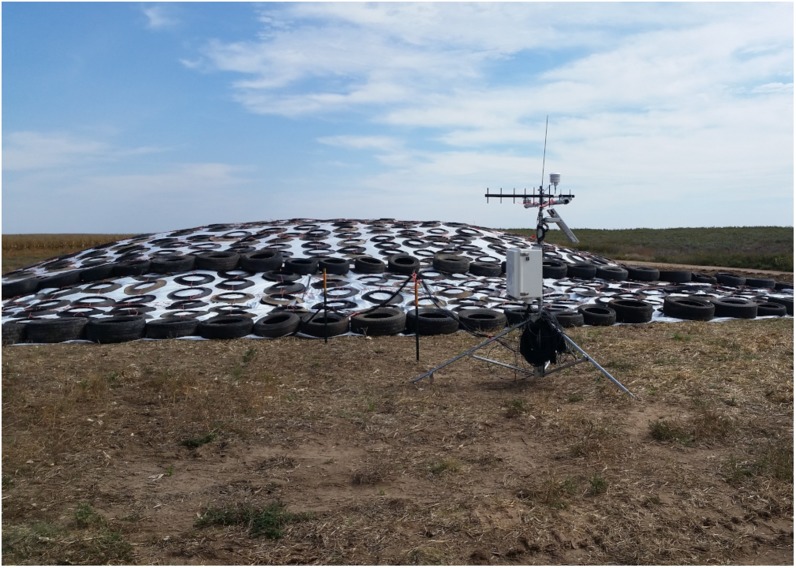
Covered drive over storage pile.

An alternative approach to feedstock supply logistics systems that rely on baling biomass is to adopt the commonplace practices of the forage industry. Wet, anaerobic storage systems (i.e., ensiling) are an alternative to dry storage and have consistently and successfully demonstrated biomass preservation in long term storage for livestock feed and forage. Wet biomass logistics systems have been proposed for corn stover, primarily to address the concern of catastrophic loss of corn stover stacks to fires (Wendt et al., 2018a, b). Wet logistics systems are based on forage chopping herbaceous biomass in the field at moisture contents between 40 and 65% (wet basis), transporting the chopped biomass in silage trucks, and utilizing anaerobic storage systems including silage bags, bunkers, or drive-over piles to limit oxygen and preserve biomass (Ferraretto et al., 2018). Figures 7–9 show the harvest, transport and unloading, and resulting anaerobic storage pile described in Wendt et al. (2018a). Ensiling is a common practice for corn and grasses in humid climates of the world including parts of the United States and in Europe (Muck and Kung, 2007). Over 121 million tons of corn silage were harvested in 2018 in the United States and stored for livestock forage using this approach (U.S. Department of Agriculture, 2018). Ensiled biomass can be stable for months to years if anaerobic conditions are maintained. Expected dry matter losses under best management practices range from 6 to 15% depending on storage structure, with losses as low as 3% possible (Muck and Kung, 2007; Borreani et al., 2018).

The success of ensiling relies on mechanical exclusion of air through compaction, utilization of oxygen present through respiration, and fermentation to produce organic acids and a corresponding reduction in pH (McDonald et al., 1991). Obligate aerobic microorganisms are primarily responsible for the initial consumption of oxygen through respiration, although plant respiration also plays a role (Pahlow et al., 2003). Once this oxygen has been consumed then lactic acid bacteria proliferate and produce organic acids (Pitt et al., 1985). Soluble sugars, which are commonly referred to as water soluble carbohydrates in the forage literature, serve as the energy and carbon source for the initial fermentation as well as sustained but reduced growth of lactic acid bacteria during the stable storage stage (Pahlow et al., 2003). The combination of anaerobic conditions and the presence of organic acids and corresponding low pH serve to reduce overall microbial activity in ensiled systems, and Leistner (2000) described this combination of factors to promote stability as the hurdle concept.

The soluble sugars in biomass can constitute a significant portion of biomass, and their presence is important for successful ensiling. These sugars are transported through actively growing plants, forming structural sugars as the plant grows (Cosgrove, 2005). Corn stover can contain between 4 and 12% of these soluble carbohydrates depending on the growth phase of the plant (Chen et al., 2007). Forages grasses can have a wide range of soluble carbohydrates with anywhere from 5 to 30% (McDonald et al., 1991), and up to 16.3% soluble carbohydrates have been documented in switchgrass (Dien et al., 2006). Sweet sorghum can contain up to 20% soluble carbohydrates (Rains et al., 1990). The stage of growth often determines the level of soluble carbohydrate reserves in the plant, with the levels decreasing after anthesis and as the plant sends carbohydrate reserves to the roots for wintering. Soluble carbohydrate levels in grasses have been shown to vary between 10 and 35% depending on the environmental conditions and the stage of growth (Wulfes et al., 1999). Similarly, soluble carbohydrate levels in corn stover as low as 2.5% of total mass have been present at the time of harvest and still resulted in successful preservation in ensiling (Wendt et al., 2018a). Ensuring that sufficient fermentable soluble sugars are present at the time of ensiling is necessary to support organic acid production and pH reduction. Low cost additives such as molasses or chemicals can be applied when sufficient soluble sugars are not available, as discussed in the following sections.

Dry matter loss and final pH during the ensiling process is related to the type of lactic acid bacteria present and their fermentation pathway. Lactic acid formation by homofermentative lactic acid bacteria during ensiling results from the direct conversion of glucose to lactic acid, whereas heterofermentative lactic acid bacteria convert glucose to lactic acid, acetic acid, ethanol, and CO_2_ (McDonald et al., 1991). Therefore, homolactic acid fermentation results in lowest losses of carbon and associated dry matter and is preferred during ensiling. However, acetic and propionic acids have been shown to inhibit spoilage microorganisms during aerobic exposure at the time of utilization of silage (Kung et al., 1998). Therefore, a mixture of acids is commonly desirable in ensiled biomass.

The protective effect of organic acids during preservation is based on inhibition of unwanted microorganisms. Lambert and Stratford describe the mechanism by which undissociated weak acids permeate across microbial plasma membranes and then dissociate into protonated hydrogen molecules and deprotonated hydroxyl groups (Lambert and Stratford, 1999). This is followed by proton pumping out of the cell, which leaves the hydroxyl group in the cytochrome and thus lowers the internal cell pH (Lambert and Stratford, 1999). The low p*K*_a_ of lactic acid (3.78) makes this the preferred organic acid for stability compared to acetic acid (p*K*_a_ = 4.75) or butyric acid (p*K*_a_ = 4.82). Lactic acid dominated silages tend to have a pH near 3.7–3.9, and thus there is an overall increase in the level of undissociated acids outside of cell walls at lower pH values.

Degradation as a result of oxygen exposure in ensiling is a significant risk for these storage systems. Oxygen exposure is present during the formation and deconstruction of anaerobic storage piles. Delayed sealing or covering in ensiling has been shown to encourage the consumption of soluble carbohydrates by aerobic bacteria, yeast, and fungi (Henderson and McDonald, 1975; Pahlow et al., 2003). This results in not only less of this carbon source being available for lactic acid bacteria but also competition between lactic acid bacteria and clostridia. Clostridia produce butyric acid in silage, which is associated with higher dry matter loss in storage and lower consumption of the forage by ruminants (Muck and Kung, 2007). Clostridia spores can be passed into milk and can lead to contamination in milk and the products that are made from milk including cheese (Drouin and Lafrenière, 2012). This issue is of lower concern for bioenergy systems because pretreatment generally occurs at temperatures that can deactivate spores such that they are not passed into the fermentation process. However, the higher dry matter loss as a result of oxygen exposure is a concern for bioenergy systems due to the loss of convertible carbon to the atmosphere.

Corn stover for bioenergy production is available at the time of grain harvest and accordingly contains lower initial moisture contents and lower soluble sugars compared to feedstock dedicated for forage (Pordesimo et al., 2004, 2005). This presents a challenge when ensiling corn stover because the reduction of water corresponding increases the interstitial oxygen that must be either mechanically removed or biologically consumed in order to establish conditions that favor fermentation. Similarly, insufficient soluble carbohydrates for fermentation ultimately result in lower organic acid production. Despite these challenges, Shinners et al. and Wendt et al. both demonstrated that low-moisture ensiling (∼40% moisture, wet basis) was possible, with <5% loss was experienced over 6 months in covered, drive-over storage piles (Shinners et al., 2011; Wendt et al., 2018a). Similarly, ensiled corn stover has demonstrated slight pretreatment in ensiled storage conditions (Darku et al., 2010; Essien and Richard, 2018). Therefore, ensiling provides a solution for biomass to be stored in a stable format and utilized in bioenergy conversion systems throughout a calendar year notwithstanding the biomass being seasonally available.

Long-term wet, anaerobic storage has been shown not only to stabilize biomass but can also provide an environment to begin depolymerization of structural components, such as lignin and hemicellulose, a benefit that could help to lower conversion costs for high moisture feedstock. The high moisture environment provides an environment that enables biological and chemical reactions to occur. The pH of typical ensiled material is in the range of 3.5–4.5, depending on the fermentation pathway. This pH range inhibits most growth by obligate aerobic bacteria, yeast, and fungi, and even lactic acid bacteria have reduced activity at pH levels below 4 (Venkatesh et al., 1993). However, organisms that are active may be producing enzymes that can liberate the carbohydrates from the biomass and support their growth. Fructan hydrolases produced from the ensiled plants themselves (Ould-Ahmed et al., 2017) or by select lactic acid bacteria strains that can create fermentable sugar monomers from polysaccharides (Merry et al., 1995; Muck and Kung, 2007). This may occur in anaerobic storage systems even with low degradation rates. Gusovius et al. (2019) correlated the reduction of fiber size in hemp to dissolution of the middle lamella by microbial activity in anaerobic storage. Similarly, delamination in the middle lamella in pine has also been documented as a result of fungal treatment (Goodell et al., 2017). Further investigation is necessary to understand the role of long-term storage to influence cell walls and related structural integrity of biomass.

Despite the multiple benefits of wet anaerobic systems for corn stover in promoting stability in long term storage, prior research has been unable to close the cost gap between wet systems and their lower cost dry counterparts. The primary drawback of wet systems for corn stover is that the moisture in the biomass as well as the bulk, chopped format makes handling this biomass more costly than handling dry, baled biomass. For example, prior research has shown that transportation costs double for chopped corn stover compared to baled stover as a result of reduced bulk density compared to baled biomass (Wendt et al., 2018b). However, the size reduction that can be accomplished during forage chopping that is used in wet logistics systems can reduce both harvest and collection costs as well as the cost of further size reduction during preprocessing. Harvest and collection costs were reduced from $21 Mg^–1^ in a bale-based logistics system to less than $16 Mg^–1^ in a wet logistics system (Wendt et al., 2018b). Likewise, size reduction during forage chopping is capable of reducing particle size geometric mean to 5–10 mm (Lisowski et al., 2017), whereas baled logistics systems for corn stover rely on one to two steps of size reduction with a 6 mm screen during preprocessing. However, wet anaerobic storage costs are higher more than its baled counterpart. Field-side storage costs for baled corn stover are estimated to range between $5 and $18 Mg^–1^, while anaerobic storage of corn stover in piles is estimated to cost between $15 and $22 Mg^–1^ (2015 US dollars, Vadas and Digman, 2013; Wendt et al., 2017, 2018b). Additional research is necessary to identify approaches that willo address the cost barrier of wet anaerobic storage compared to baled storage.

### Storage Selection Based on Feedstock Type

Feedstock type and harvest scenario both impact the most suitable long-term storage approach. Table 1 lists the herbaceous crop residues and energy crops identified in the Billion Ton report (Langholtz et al., 2016) and the most common storage approach utilized for them. Residues that are harvested based on timing of the grain harvest are generally lower moisture content and compatible with baled storage; these include the straws and grain sorghum stubble. Energy crops including switchgrass and miscanthus are generally harvested after senescence and subsequently stored in baled formats. However, harvest of these plants is not dependent on a primary commodity crop and the timing can be flexible such that anaerobic wet storage could be compatible with these crops. Crops that are high moisture at the time of storage including energy cane and sugarcane bagasse are best suited for wet storage systems. As discussed previously, corn stover is often stored in dry, baled formats, but challenges with achieving the desired moisture content for stability are inherent to this crop and provide an opportunity for wet storage to address this challenge. However, long-term wet storage operation is one of the unit operations in the feedstock logistics operations that can be used to improve the quality of the corn stover with the aim of reducing downstream processing requirements for conversion to fuels and chemicals. The following sections describe approaches that have or could be used to facilitate this reduction in recalcitrance.

**TABLE 1:** Herbaceous crop residues and energy crops identified in the Billion Ton study linked to their common storage method.

**Biomass type**	**Dry storage**	**Wet storage**
Barley straw	x	
Corn stover	x	x
Energy cane	x	x
Grain sorghum stubble	x	
Miscanthus	x	x
Rice straw	x	
Sugarcane bagasse		x
Switchgrass	x	x
Wheat straw	x	

### Storage Amendments

The application of amendments to biomass to promote stability prior to anaerobic storage is commonplace in the forage industry. The goal of these amendments is to promote the fastidious formation of a low pH environment that result in stable storage and maintain desirable qualities for forage (Muck et al., 2018). Amendments may include acids or alkali applied directly to the biomass or microbial amendments to encourage a specific fermentation pathway, and either of these can be effective at reducing storage losses. Storage amendments are so commonplace that forage choppers are often equipped with sprayers that can apply liquid inoculants during harvest. The following section describes some of the primary amendments that have been used over the last century for forage silage and may have applicability for bioenergy systems.

#### Microbial Amendments

Lactic acid bacteria are commonly added to silage during harvesting to promote the proliferation of these organisms and thus more rapid fermentation during ensiling (Muck and Kung, 2007). Homofermentative lactic acid bacteria that produce primarily lactic acid have demonstrated reduced aerobic stability upon removal from storage compared to the acetic acid containing biomass produced by heterofermentative lactic acid bacteria (Muck and Kung, 2007; Muck et al., 2018). A wide range of microbial inoculants are available commercially, and they generally contain a mixture of bacterial species to improve the palatability of the feedstock for livestock (Muck et al., 2018). Anaerobic storage with microbial inoculants has been suggested to positively influence performance in bioenergy conversion systems. The combination of high-moisture storage with bacterial inoculants have been demonstrated to increase sugar release in wheat and rice straw, corn stover and corn silage, and forage sorghum (Linden and Murphy, 1990; Henk and Linden, 1996; Oleskowicz-Popiel et al., 2011).

Enzymes have also been added to silage in order to increase the level of soluble carbohydrates for consumption by lactic acid bacteria (Kung et al., 1991; Kung, 1998). Common enzymes include cellulases, xylanases, and pectinases, and most are applied in combination with a lactic acid bacteria inoculant that can utilize the sugars released enzymatically (Muck et al., 2018). Organisms that produce ferulic acid esterase have also been added to silage with mixed success in improving digestibility of livestock feed (Lynch et al., 2015). Enzymes also have a role in bioenergy conversion systems, where depolymerization of structural hemicellulose in long-term storage could be utilized to reduce pretreatment severity at the biorefinery. Low-moisture corn stover (∼20%, wet basis) amended with xylanase increased recovery of hemicellulose-related sugars by 10% over untreated controls during long-term storage (Smith et al., 2009). A common concern when adding enzymes during long-term storage is the excessive hydrolysis of carbohydrates (Kung and Muck, 2015), which results in elevated substrate for fermentation in anaerobic storage or excessive loss upon aerobic exposure. This balance must be carefully managed based on feedstock type and utilization strategy.

#### Acidic Amendments

Organic and mineral acids have been used extensively in silage to rapidly decrease pH and preserve the nutrient content of the biomass. Virtanen used a blend of hydrochloric and sulfuric acids to preserve silage, and this work demonstrated that a pH of 4.0 was necessary to inhibit soluble carbohydrate and protein degradation along with butyric acid formation (Virtanen, 1933). Virtanen received a Nobel Prize in Chemistry in 1945 for this and delete Contribution To The Field (The Nobel Prize in Chemistry, 1945). Sulfuric acid is a strong acid and is used specifically to reduce pH, however, Virtanen recommended that it not be applied alone due to poor digestibility by rumen. Formic acid is common silage additive that is considered to reduce pH rapidly as well as provide antimicrobial effects. Formic acid is proposed to disrupt the electron transport chain by inhibiting cytochrome oxidase (Keyhani and Keyhani, 1980). While this may be desired for the suppression of spoilage microorganisms, this same mechanism has resulted in histotoxic hypoxia in farmers exposed to vapors while making silage (Liesivuori and Kettunen, 1983). It has also been noted that yeasts have a higher tolerance to formic acid treated silages than lactic acid bacteria, such that the aerobic stability of formic acid treated silages is poor (Henderson et al., 1972). Formic acid is still used as a silage additive, particularly in European countries due to the ban on antibiotics in livestock feed. However, its use is limited in the United States because it traditionally is a higher cost acid. Approaches to produce lower-cost formic acid are necessary to enable additional utilization of this acid in forage and bioenergy storage systems.

Propionic acid is a low-cost additive often used in the United States, particularly in haylage (Knapp et al., 1976). Propionic acid additives have demonstrated to reduce yeast proliferation upon removal of ensiled biomass from storage, thus increasing the aerobic stability of the biomass (Woolford, 1975). Similarly, numerous acid and acid salt combinations have been described for their preservation effect on silage during storage and upon exposure to oxygen (Muck et al., 2018). Nadaeu et al. demonstrated an improvement in aerobic stability of corn silage from 5.7 to 11.8 days for biomass that entered storage after treatment with a combination of formic, propionic, benzoic, and sorbic acids (Nadeau et al., 2011). Acid salt combinations including potassium sorbate, sodium benzoate, and sodium nitrite have also shown to increase aerobic stability in corn silage (Da Silva et al., 2015). Perennial grasses, including switchgrass, have been successfully preserved in high-moisture storage amended with mineral acid and experienced up to 17% improvement in cellulose conversion to ethanol (Williams and Shinners, 2012). In summary, acids have demonstrated as effectiveness as a direct approach in improve ensiling performance and aerobic stability of biomass upon utilization. Further knowledge on the effect of these treatments to improve performance in bioconversion to fuels and chemicals will increase their utilization in commercial biorefineries.

#### Alkaline Amendments

Alkaline treatments have been used for stabilizing wet harvested biomass by creating a basic environment which can restrict unwanted fermentation. Anhydrous ammonia has been applied to forage for over 50 years to improve nitrogen levels and prevent proteolysis and deamination in forage, which improves the quality of the biomass for livestock feed (Huber and Santana, 1972; Huber et al., 1979). Anhydrous ammonia has been demonstrated to raise pH and decrease lactic acid formation during the initial days of ensiling as well as decrease protein degradation in long-term anaerobic storage (Johnson et al., 1982).

Calcium oxide, or lime, has been used as an additive for biomass with the dual aim of improving storage stability as well as to impact thermochemical conversion performance (Xiong et al., 2017; Bozaghian et al., 2018). Calcium oxide (CaO) reacts with water to produce calcium dihydroxide [Ca(OH)_2_], which then reacts with CO_2_ to form calcium carbonate (CaCO_3_). Calcium carbonate is understood to act as a sorbent and reacts with other inorganics including silica and sulfur during thermochemical conversion (Wang et al., 2016), which increases the melting temperature of the resulting inorganic complex and thus reduces undesirable slagging on reactor surfaces and catalysts (Bozaghian et al., 2018). Calcium oxide treatment of reed canary grass was shown to increase pH to greater than 9 in biomass containing 35–65% moisture, which is desirable to reduce proteolytic organisms but not sufficiently high such that protein degradation occurred. In this study the 35% moisture content biomass exhibited stable aerobic storage over 90 days due to the combined effect of initial increased pH and reduction of moisture through drying (Xiong et al., 2017), however, higher moisture contents levels resulted in storage losses up to 30% and the subsequent reduction of pH levels to 8–9 likely as a result of liberation of acetyl side chains from the hemicellulose. Similarly, lime has been applied to poplar over a 12 week period to enhance the solubilization of lignin though oxidation and deacetylation of hemicellulose through hydrolysis in order to improve the digestibility of wood in enzymatic hydrolysis (Rocio et al., 2011).

Sodium hydroxide has been assessed for use during storage to reduce biomass recalcitrance, and the advantage of this alkali above lime is that it is readily soluble. Sodium hydroxide has been used to improve the digestibility of wheat and barley straws for livestock feed by reducing lignin content (Chesson, 1981; Lindberg et al., 1984). Sodium hydroxide treatment during 1–3 days of storage has also been applied to corn stover at 80% moisture content (wet basis) in order to increase biogas yields in anaerobic digestion, and these studies have indicated that hemicellulose is most susceptible to short-term sodium hydroxide exposure as a result of removal of acetyl groups (Pang et al., 2008; Zheng et al., 2009; Zhu et al., 2010). Cui et al. investigated the use of sodium hydroxide treatment during 90-day ensiling of corn stover in plastic bags at moisture contents ranging from 45 to 75% moisture (wet basis) (Cui et al., 2012). This study showed that lignin and cellulose degradation was complete within 5 days of storage but that xylan degradation continued over the 90-day storage period; however, significant dry matter loss of 13–21% occurred during the storage period. An increase in acetic acid levels was observed during the first 15 days of storage, and subsequent reduction of structural acetate after this period is consistent with the dry matter loss experienced. Similarly, glucose and xylose yields were reduced in samples that experienced 90 days of storage compared to 5 and 12 days of storage. This study shows the importance of maintaining stable storage conditions when combining sodium hydroxide with long term storage.

Alkali treatments have shown to reduce chemical recalcitrance of biomass to deconstruction and are the state-of-the-technology for cost-competitive biochemical conversion of carbohydrate and lignin monomers to biofuels (Chen et al., 2016; Davis et al., 2018). However, these high-severity treatments require significant alkali loading during short thermal residence times in order to be efficient at the biorefinery scale. Anaerobic storage offers the opportunity to allow the deacetylation reactions to occur over a longer residence time with the added benefit of protecting biomass from uncontrolled dry matter loss. As discussed in this section, alkali treatment has demonstrated reduced recalcitrance in terms of improved digestibility for rumen. However, the combination of well-preserved biomass resulting from anaerobic storage and alkali treatment have not yet been applied in relation to both physical and chemical preprocessing to form convertible carbohydrate monomers for bioenergy systems.

### Storage Systems Linked to Conversion

The impacts of long-term storage are an important variable to consider in the conversion of biomass resources to fuels and chemicals. The conditions experienced during storage and resulting biochemical changes in cells can positively or negatively impact conversion potential. For example, corn stover that had experienced significant aerobic degradation (30% loss of dry matter) was shown to have a significant shift in structural to soluble xylan but no change in structural glucan (Wendt et al., 2018a). However, after either dilute acid or dilute alkaline treatment the efficiency of enzymatic hydrolysis to depolymerize glucan was increased in the aerobically degraded biomass; this suggests that the loss of hemicellulose in storage resulted a slight pretreatment effect. Dilute acid and dilute alkaline pretreatments have been applied to anaerobically stored biomass as well with success (Wendt et al., 2018a), and in this case dilute alkali treatment was effective in showing an increase in carbohydrate release after anaerobic storage. Limited data on deacetylation pretreatment is available for corn stover, but alkali groups in hemicellulose hydrolyzed during storage should positively impact deacetylation. Additionally, organic acids produced during anaerobic storage may serve as catalyzing agents during pretreatment including during steam explosion (Liu et al., 2013) or hot water extraction (Ambye-Jensen et al., 2018; Essien and Richard, 2018). However, ammonia fiber expansion pretreatment is primarily performed prior to long term storage because it results in a shelf-stable format. Additional insight is needed to understand how long-term storage can be used to enhance deconstruction based on each biomass type and each pretreatment chemistry.

## Conclusion and Future Directions

Long-term storage of biomass is a reality for any agricultural system and is a key unit operation for bioenergy systems. However, the costs necessary to produce stable storage conditions are often misaligned with the pressures of producing biofuels that are competitive with their fossil counterparts. Focus on multiple research directions can address this cost disparity and should include (1) understand how baled biomass systems can provide protection from moisture and related physical and microbial losses, (2) application of how wet, anaerobic systems commonly used in forage might be used to overcome the cost barrier that currently makes them less attractive for bioenergy systems, and (3) an enhanced understanding of how these storage systems may affect biomass recalcitrance and subsequent conversion to fuels or chemicals. There is also potential to shift the focus of long-term storage from a cost center to a value-added operation such that bioconversion, energy balances, and sustainability are positively impacted. Securing the storage operation of the feedstock logistics and supply chain will be a key component to making the bioeconomy a reality.

## Author Contributions

LW drafted the manuscript with contributions from HZ. LW and HZ revised the manuscript and prepared figures. All authors approved the manuscript for publication.

## Conflict of Interest

The authors declare that the research was conducted in the absence of any commercial or financial relationships that could be construed as a potential conflict of interest.
